# Decadal acidification in Atlantic and Mediterranean water masses exchanging at the Strait of Gibraltar

**DOI:** 10.1038/s41598-019-52084-x

**Published:** 2019-10-29

**Authors:** Susana Flecha, Fiz F. Pérez, Akihiko Murata, Ahmed Makaoui, I. Emma Huertas

**Affiliations:** 1 Instituto Mediterráneo de Estudios Avanzados (CSIC-UIB), Miquel Marquès 21, 07190 Esporles, Spain; 2 Instituto de Investigaciones Marinas (CSIC), Eduardo Cabello 6, 36208 Vigo, Spain; 3 Research &Development Center for Global Change (JAMSTEC), 2-15 Natsushima-cho, 2370061 Yokosuka, Japan; 4 Institut National de Recherche Halieutique (INHR), 2, BD Sidi Abderrahmane, 20100 Casablanca, Morocco; 5 Instituto de Ciencias Marinas de Andalucía (CSIC), Polígono Río San Pedro s/n, 11519, Puerto Real, Cádiz Spain

**Keywords:** Marine chemistry, Environmental impact

## Abstract

Seawater pH is undergoing a decreasing trend due to the absorption of atmospheric CO_2_, a phenomenon known as ocean acidification (OA). Biogeochemical processes occurring naturally in the ocean also change pH and hence, for an accurate assessment of OA, the contribution of the natural component to the total pH variation must be quantified. In this work, we used 11 years (2005–2015) of biogeochemical measurements collected at the Strait of Gibraltar to estimate decadal trends of pH in two major Mediterranean water masses, the Western Mediterranean Deep Water (WMDW) and the Levantine Intermediate Water (LIW) and assess the magnitude of natural and anthropogenic components on the total pH change. The assessment was also performed in the North Atlantic Central Water (NACW) feeding the Mediterranean Sea. Our analysis revealed a significant human impact on all water masses in terms of accumulation of anthropogenic CO_2_. However, the decadal pH decline found in the WMDW and the NACW was markedly affected by natural processes, which accounted for by nearly 60% and 40% of the total pH decrease, respectively. The LIW did not exhibit a significant pH temporal trend although data indicated natural and anthropogenic perturbations on its biogeochemical signatures.

## Introduction

The atmospheric concentration of carbon dioxide (CO_2_) has markedly increased from the beginning of the industrial era due to the release of carbon from combustion of fossil fuels, deforestation and large changes in land-use activities. Anthropogenic emissions in particular, became the dominant source of CO_2_ to the atmosphere around the mid XX century and continue rising at present^1^. The global ocean plays a relevant role mitigating this rise through the absorption of around a quarter of the emissions^1^. Recently, the oceanic sink for anthropogenic CO_2_ (*C*_ANT_) over the period 1994–2007 has been estimated to represent 31% of the global emissions^2^. Hence, the oceanic withdrawal of atmospheric CO_2_ alleviates the greenhouse effect, but there is also a downside, as the CO_2_ absorbed by the ocean changes the chemistry of seawater by lowering its pH and the carbonate ion (CO_2_^3−^) levels. The chemical reactions derived from the uptake of CO_2_ are generally termed ocean acidification^3^ and threaten the overall structure of marine ecosystems at a planetary scale^4^.

**Figure 1:**
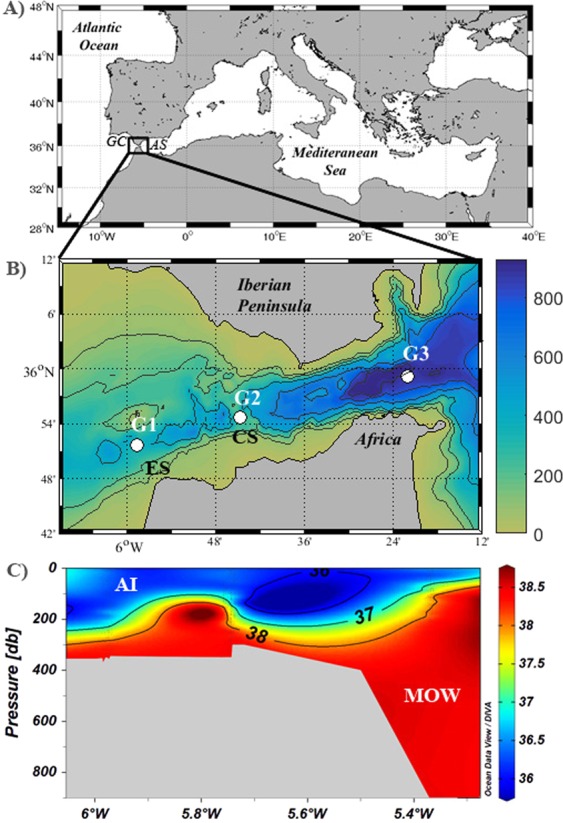
Study area. (**A**) Location of the Strait of Gibraltar, Gulf of Cadiz (GC) and Alboran Sea (AS); (**B**) Position of GIFT stations (G1, G2 and G3), Camarinal sill (CS) and Espartel sill (ES). Bathymetry (m) in the channel is indicated with the color contour; (**C**) Spatial distribution of salinity based on archetypal values. AI and MOW stand for Atlantic inflow and Mediterranean Outflow Water respectively. Ocean Data View software (Schlitzer, R., Ocean Data View, odv.awi.de, 2018) was used to display data.

Ocean acidification trends have been already documented through multi-decadal measurements in open ocean marine observatories^5^. It has been also suggested that certain basins, such as marginal seas, will be more impacted by the phenomenon than others^6^. This is the case of the Mediterranean Sea (MedSea), whose high sensitivity to acidification has been attributed to particular biogeochemical features and water circulation patterns^7,8^. The high alkalinity of Mediterranean waters and an active overturning circulation result in both an increased absorption of atmospheric CO_2_ and an intensified carbon transport from the surface to the ocean interior^6,9,10^. Moreover, *C*_ANT_ is imported continuously from the North Atlantic through the Strait of Gibraltar (SoG, Fig. 1A)^10,11^, leading to its accumulation in the basin at a long term.

The MedSea is indeed experiencing a decrease in pH although different declining rates have been reported^9,10,12–14^. Some works have provided a total pH decline between −0.055 and −0.156 pH units^9,12^ whereas other studies reported values ranging from −0.005 to −0.06 pH^10,13^. These discrepancies are based on the simulation of the quantity of *C*_ANT_ that is taken up by Mediterranean water masses. This amount cannot be measured directly because it cannot be distinguished from the much larger natural carbon background and it has to be calculated through indirect approaches. The estimation of *C*_ANT_ using methods that combine observable physical and biogeochemical variables or models result then in different acidification rates.

However, seawater pH also varies “naturally” as a consequence of physical and biological processes, such as ocean circulation mechanisms and/or changes in ecosystem metabolism^15–17^. In fact, the pH decline due to biology in Atlantic water masses is of the same order of magnitude than the pH decrease caused by *C*_ANT_ absorption^15^. Similarly, in the Northeast Pacific coastal ocean, long-term ocean acidification is modulated by the pH variability driven by ecosystem metabolism^17^. Hence, the accurate assessment of the actual extent of ocean acidification requires the identification of the fraction of the total pH change that is purely due to natural processes and discriminate it from the anthropogenic fraction. This analysis is particularly relevant in the MedSea where biogeochemistry is closely linked to water circulation patterns^18,19^.

In the current study we examined the relative contribution of both *C*_ANT_ uptake and biology on temporal pH trends in two major Mediterranean water masses, the Western Mediterranean Deep Water (WMDW) and the Levantine Intermediate Water (LIW). They are originated in distant regions, such as the Gulf of Lions located in the Western Mediterranean and the Levantine basin in the Eastern Mediterranean, respectively. Nevertheless, both water masses can be easily tracked in the SoG by their characteristic thermohaline signatures^13,20^.

More specifically, ocean circulation in the MedSea starts with the entry of surface water from the Atlantic Ocean through the SoG, which flows towards the east while gaining salinity and density. In the Levantine basin the density has reached such an extent that the water sinks to form the LIW, which flows back into the opposite direction in a few 100 m depth. In the Gulf of Lions, high salinity waters cool down regularly in winter reaching enhanced densities and forming the WMDW, with the highly saline LIW acting as a preconditioner for deep water formation^21,22^. Both water masses leave jointly the MedSea as part of a dense (salinity >38) Mediterranean Outflow Water (MOW) that occupies the bottom layer in the SoG (Fig. 1C). The MOW is compensated by the surface eastward warmer (and less salty) Atlantic Inflow (AI, Fig. 1C) that is mainly constituted by the North Atlantic Central Water (NACW). The MOW and AI are clearly distinguishable within the channel by their different physical and biogeochemical properties, such as potential temperature, salinity, alkalinity, dissolved oxygen, inorganic nutrients and trace gases, as shown by measurements at the Gibraltar Fixed Time series (GIFT, Fig. 1B)^11,23,24^.

Here, we present the first (to our knowledge) measurement-based temporal evolution of the carbon system parameters in water masses exchanging through the SoG using observations collected between the years 2005–2015, with special emphasis in decadal trends of pH. The anthropogenic (*pH*_*nobio*_) and natural (*pH*_*bio*_) components of the decadal pH change were subsequently obtained in order to identify the relative contribution of both drivers on the pH variability observed in each water mass.

## Materials and Methods

### Dataset

Data were collected periodically at three stations that form the GIFT time series, G1 (5°58.60W, 35°51.68N), G2 (5°44.75W, 35°54.71N) and G3 (5°22.10W, 35°59.19N) (Fig. 1B), during 26 campaigns conducted over the decade 2005–2015 (Table 1). In 8 cruises (denoted as CARBOGIB in Table 1), 4 more stations of a North-South grid perpendicular to the GIFT section were sampled, (5°44.04W, 35°52.67N), (5°44.36W, 35°53.50N), (5°45.33W, 35°56.56N) and (5°45.56W, 35°58.53N). Even though several analysis have allowed to conclude that monitoring at the GIFT is effective to fully resolve the vertical and horizontal structure of the water column in the area^11,23,24^, data acquired in the perpendicular leg have been also included in our assessment (Table 1). A total of 5,145 measurements have been used.

**Table 1:** Campaigns conducted in the Strait of Gibraltar from 2005 to 2015 that provided data used in this study. Temporal coverage, levels sampled and measurements used to perform the assessment are also indicated. More details regarding sampling design and strategy are indicated in the text.

Campaign ID	Date	Ship	Number of depths/measurements
CARBOGIB1	02–05 May, 2005	RV Al Amir Moulay Abdellah	40/304
GIFT 0605	09–10 May, 2005	BO García del Cid	14/112
CARBOGIB3	12–14 December, 2005	RV Al Amir MoulayAbdellah	37/291
CARBOGIB4	20–22 March, 2006	RV Al Amir MoulayAbdellah	40/300
CARBOGIB5	21–23 May, 2006	RV Al Amir MoulayAbdellah	40/313
GIFT1106	23–24 November, 2006	BO García del Cid	15/120
CARBOGIB6	13–15 December, 2006	RV Al Amir MoulayAbdellah	37/286
CARBOGIB7	07–08 May, 2007	RV Al Amir MoulayAbdellah	37/256
CARBOGIB8	04–05 July, 2007	RV Al Amir MoulayAbdellah	37/282
CARBOGIB9	06–07 November, 2007	RV Al Amir MoulayAbdellah	34/244
SESAME1	13–14 April, 2008	BO Regina Maris	23/176
SESAME2	27–28 July, 2008	BO Regina Maris	27/193
SESAME3	25–26 September, 2008	BO García del Cid	25/198
P3A2_1^ST^_LEG	12–13 October, 2008	RV Hespérides	21/168
P3A2_2^ND^_LEG	20–21 October, 2008	RV Hespérides	21/166
GIFT0409	24 April, 2009	BO Sarmiento de Gamboa	15/119
GIFT0609	1 July, 2009	BO Sarmiento de Gamboa	15/119
GIFT0909	25 September, 2009	BO Sarmiento de Gamboa	26/207
GIFT1109	24 November, 2009	BO Sarmiento de Gamboa	25/200
GIFT0711	26 July, 2011	BO García del Cid	26/185
GIFT0811	4–5 August, 2011	BO Cornide de Saavedra	21/159
GIFT1111	10 November, 2011	BO García del Cid	18/142
GIFT0212	27–28 February, 2012	BO García del Cid	22/175
GIFT0513	19 May, 2013	RV Hespérides	18/143
GIFT1114	3 November, 2014	BO Socib	18/144
GIFT0615	6 June 2015	BO Socib	18/143

Data acquisition procedure was identical for all campaigns. A temperature and salinity profile was obtained with a Seabird 911Plus CTD probe. Seawater was subsequently collected for biogeochemical analysis using Niskin bottles immersed in an oceanographic rosette platform at variable depths (from 5 to 8 levels) depending on the instant position of the interface between the AI and MOW that was identified by the CTD profiles

### Biogeochemical analysis

The biogeochemical variables considered in this study have been pH in total scale at 25 °C (pH_T25_), total alkalinity (A_T_), Dissolved Oxygen (DO) and inorganic nutrients (phosphate, PO_4_^3−^, nitrate, NO_3_^−^ and Silicate, SiO_4_^4−^).

pH_T25_ data were obtained by the spectrophotometric method with m-cresol purple as indicator^25^. Samples were taken directly from the oceanographic bottles in 10 cm path-length optical glass cells and measurements were carried out with a Shimadzu UV-2401PC spectrophotometer containing a 25 °C-thermostated cells holder^11^. Precision and accuracy of the pH measurements were ± 0.0052 and ± 0.0055 respectively, which were determined from measurements of certified reference material (CRM batches#94, #97 and #136 provided by Prof. Andrew Dickson, Scripps Institution of Oceanography, La Jolla, CA, USA).

For further analysis of the influence of the age of water masses on the pH evolution, the theoretical seawater pH values that would result exclusively from the uptake of atmospheric CO_2_ were also calculated with the CO2SYS program^26^ using temperature, pressure, A_T_, pH_T25_ and inorganic nutrients as inputs parameters. Atmospheric CO_2_ levels were taken from the Izaña Observatory (Meteorological State Agency of Spain, AEMET, https://www.esrl.noaa.gov/)

Samples for A_T_ analysis were collected in 500-ml borosilicate bottles, and poisoned with 100 μl of HgCl_2_-saturated aqueous solution and stored until measurement in the laboratory. A_T_ was measured by potential titration^27^ with a Titroprocessor (model Metrohm 794). Precision and accuracy of A_T_ measurements were ±0.9 and ±5.4 μmol kg^−1^ respectively, which were determined from measurements of 54 CRMs of the above mentioned batches.

Concentration of dissolved inorganic carbon *c*(DIC) and partial pressure of dissolved CO_2_ (*p*CO_2_) were calculated using CO_2_SYS with the dissociation constants for carbon^28,29^ and sulphate^30^.

DO concentration [*c*(DO)] was determined through automated potentiometric modification of the original Winkler method using the Titroprocessor. Upon collection, flasks were sealed, stored in darkness and measured within 24 h. The error of measurements was ±2 μmol kg^−1^ (n = 115). Apparent Oxygen Utilization (AOU) values were calculated with the solubility equation^31^.

Water samples (5 mL, two replicates) for inorganic nutrients determination were taken, filtered immediately (Whatman GF/F, 0.7 μm) and stored frozen^32^ for later analyses in the shore-based laboratory. Nutrients concentrations were measured with a continuous flow auto-analyzer (SkalarSan ++215) using standard colorimetric techniques^33^. Analytical precisions were always better than ±3%.

### Anthropogenic carbon estimation

*C*_ANT_ concentration was estimated using the back-calculation technique^34^ by applying the following equation:

1 $${C}_{{\rm{ANT}}}(\Delta {C}^{\ast })={C}_{T}-\frac{c(AOU)}{{R}_{C}}-1/2(\Delta {A}_{T}+\frac{c(AOU)}{{R}_{N}})-{C}_{T278}^{\circ }+\,\Delta {C}_{dis}$$

where the stoichiometric coefficients R_C_ (−ΔO_2_/ΔC) = 1.45 and R_N_ (−ΔO_2_/ΔN)=10.6 were used^35^. *c*(AOU)/R_C_ corresponds to the DIC increase due to organic matter oxidation, 1/2(ΔA_T_ + *c*(AOU)/R_N_) accounts for the DIC change due to CaCO_3_ dissolution–precipitation and ΔA_T_ = A_T_ − A_T_° is the total alkalinity variation since the water mass was formed. Preformed alkalinity (A_T_°) and the disequilibrium term that stands for the air–sea CO_2_ difference (ΔC_dis_) were calculated for each water sample from the mixing proportion of the different water masses. This was carried out by an extended optimum multiparameter analysis (eOMP)^36,37^. The A_T_° type values for the NACW were calculated using the approach proposed by^38^ while those proposed by^39^ and^40^ were used in the case of the MOW, by considering existing CFC and CO_2_ exchange data from the Mediterranean basin, following a previous study^37^. C°_T278_ represents the *c*(DIC) in equilibrium with the preindustrial atmospheric CO_2_ molar fraction of 278 ppm and was calculated with the proper dissociation constants^28–30^. (ΔC_dis_) for both NACW and MOW were considered to be −12 ± 5 μmol kg^−1^ and 0 ± 5 μmol kg^−1^, respectively^11,41^. Uncertainty associated to *C*_ANT_ calculation was estimated through an error propagation analysis, resulting in ±5.0 μmol kg^−1^.

An additional OMP analysis was performed to resolve the water masses structure within the MOW and obtain biogeochemical parameters in the LIW and the WMDW. Potential temperature and salinity chosen as end members for water masses characterization were 13.22, 12.8 °C and 38.56, 38.45 for the LIW and the WMDW respectively^13^.

### Calculation of archetypal concentrations

In order to evaluate the decadal variation of pH_T25_, *C*_ANT_, *c*(AOU), *c*(DIC) and *p*CO_2_ in water masses at the SoG, annual archetypal concentrations were calculated, which correspond to the mixing-weighted average concentrations of a single parameter in a particular water mass^42^. The archetypal concentrations of a parameter *N* in a water mass *i* can be obtained by the equation:

2 $$ < {N}_{i} > =\frac{{\sum }_{j}{x}_{ij}{N}_{j}}{{\sum }_{j}{x}_{ij}}$$

where *N*_*j*_ is the concentration of *N* in sample *j*, and *x*_*ij*_ is the fraction of the water mass *i* in the sample *j*. Here, *x*_*ij*_ of each water mass present in the SoG was obtained by the OMP analysis. Standard deviation (SD) of archetypal concentrations was calculated through the formula:

3 $$S{D}_{{N}_{i}}=\frac{\sqrt{{\sum }_{j}{x}_{ij}{({N}_{j}-\langle {N}_{i}\rangle )}^{2}}}{{\sum }_{j}{x}_{ij}},$$

### Anthropogenic and natural components of pH change

The contribution of anthropogenic and natural drivers affecting total pH variation in each water mass was estimated by calculating the effect of non-biological processes on pH_T25_ changes (*pH*_nobio_), extracting the biological contribution as follows:

4 $$p{H}_{{\rm{nobio}}}=p{H}_{T25}+c(AOU)\,\ast \,{r}_{{O}_{2}:{C}_{org}}\,\ast \,\Delta pH,$$

where ${r}_{{O}_{2}:{C}_{org}}$ is the stoichiometric ratio^35^ and Δ*pH* was given an annual average constant value of 0.002^5^, assuming that surface seawater is in equilibrium with the global mean rate of atmospheric CO_2_ increase.

### Determination of temporal trends and Statistics

Considering the annual archetypal concentrations of each parameter during the 11 years period, annual variation trends were determined by ordinary linear regression and standard errors and 95% confidence intervals were calculated for the slopes of the regressions.

## Results

### Biogeochemistry in waters of the SoG: decadal spatial distribution

**Figure 2:**
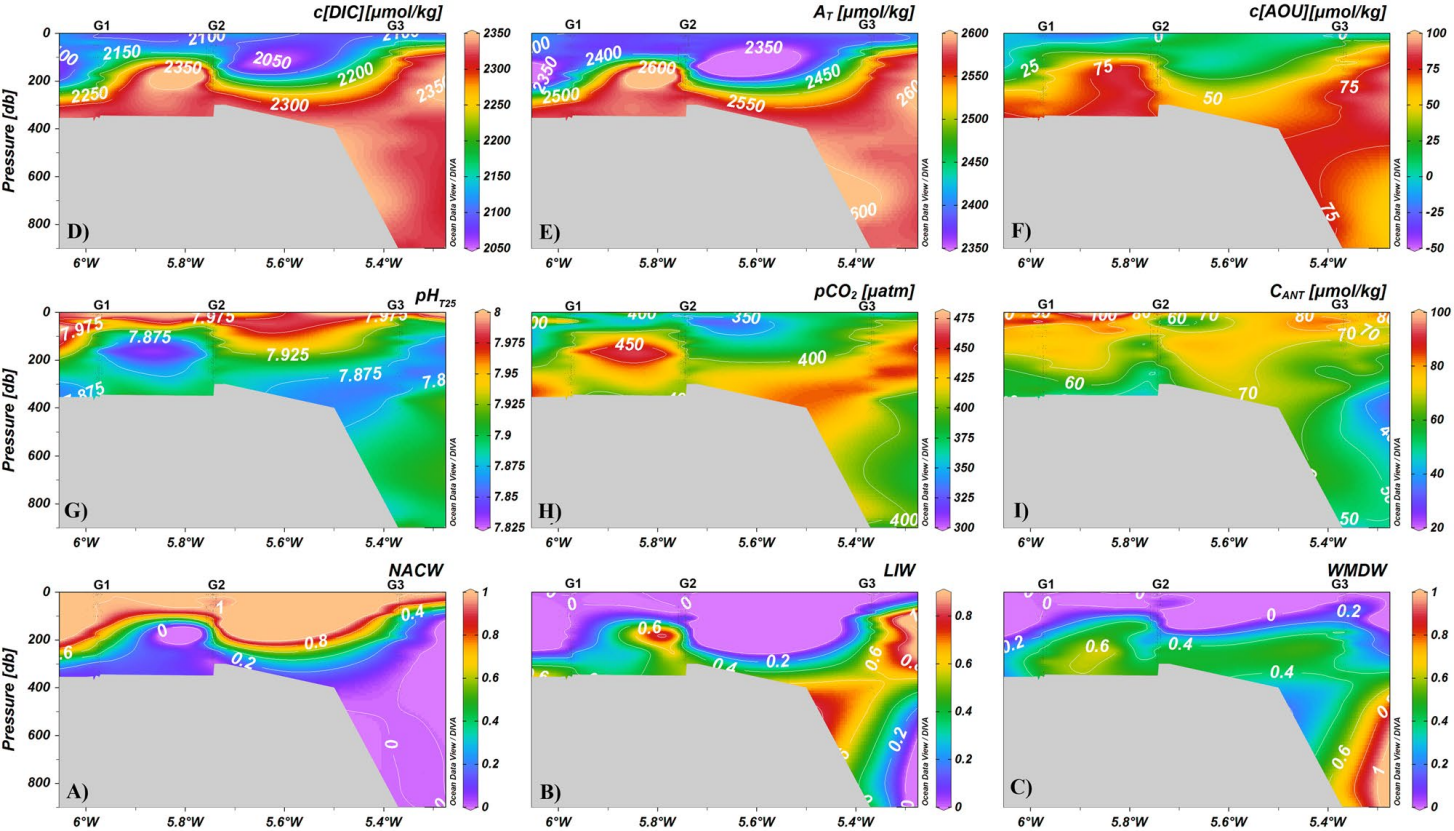
Water masses and biogeochemistry in the Strait of Gibraltar. Fraction (%) of the different water masses present in the area (panels A–C) and spatial distribution of decadal averaged concentrations of (**D**) c(DIC) (μmol kg^−1^); (**E**) A_T_ (μmol kg^−1^); (**F**) c(AOU) (μmol kg^−1^); (**G**) pH_T25_; (**H**) *p*CO_2_ (μatm) and (**I**) *C*_ANT_ (μmol kg^−1^). NACW, LIW and WMDW correspond to North Atlantic central Water, Levantine Intermediate Water and Western Mediterranean Deep Water, respectively. Ocean Data View software (Schlitzer, R., Ocean Data View, odv.awi.de, 2018) was used to display data.

Water circulation in the SoG is illustrated in Fig. 2(A–C) where the decadal averaged proportions of the water masses exchanging through the channel have been plotted. The eastward Atlantic inflow (represented here by the fraction of the NACW) invariable occupied the upper layer whereas Mediterranean waters (LIW and WMDW) flew towards the west in depth (Fig. 2A–C). However, the depth and thickness of each water mass varied along the Strait, which is due to the influence of physical mechanisms that act at different temporal scales, such as tidal currents, winds, atmospheric pressure variations and circulation processes forced by bathymetry that ultimately determine the vertical position of water masses^20^. Thus, the NACW moves upwards toward the easternmost part of the channel, shifting from 200 m at station G1 to around 70 m at station G3 (Fig. 2A). Worth mentioning is the remarkable variability in water types proportion between the Espartel sill (358 m depth) and the Camarinal sill (285 m depth) (ES and CS in Fig. 1B), which is the consequence of the enhanced mixing and turbulence related to the internal hydraulic jump formed during most tidal cycles downstream of Camarinal^43–45^. Moreover, the Atlantic water accelerates in the CS (station G2) and entrains the Mediterranean layer^43^, which results in appreciable changes in the water masses proportion around this area that can be effectively resolved by the OMP analysis (Fig. 2A–C). Even though horizontal variations in the vertical position of the LIW and the WMDW can be also observed along the channel in response to hydrodynamic features, the former was present at intermediate depths and the latter occupied the bottom layer, which is particularly clear at station G3 (Fig. 2B,C).

Water masses allocation in the SoG clearly conditions the spatial distribution of the carbon system parameters within the channel, as shown by the vertical gradients of the decadal averaged concentrations (Fig. 2D–I)

The NACW exhibited the lowest averaged *c*(DIC) and a marked vertical variability (Fig. 2D), with a mean value of 2127 ± 33 μmol kg^−1^. In contrast, the LIW and the WMDW were characterized by higher and similar mean decadal DIC levels, equivalent to 2322.6 ± 12.7 μmol kg^−1^ and 2311.7 ± 18.8 μmol kg^−1^ respectively (Fig. 2D), which match previous discrete measurements reported in different regions of the Mediterranean basin^7,9^.

A_T_ also increased gradually with depth (Fig. 2E), with lower decadal averaged concentrations being found in the upper NACW (2310 ± 22 μmol kg^−1^) in relation to those in the LIW and WMDW (2570 ± 14 and 2567 ± 21 μmol kg^−1^, respectively). The similar pattern followed by A_T_ and *c*(DIC) with salinity (Figs 1C and 2D,E) is in agreement with the conservative behaviour of these parameters described in the past^11,40^.

In contrast, pH_T25_ decreased within the water column (Fig. 2G), and decadal averaged pH_T25_ values were 7.950 ± 0.043, 7.877 ± 0.015 and 7.891 ± 0.011 in the NACW, LIW and WMDW respectively (Fig. 2G). The mean values obtained in the Mediterranean water masses coincide with earlier observations in the SoG^13^ and Western Mediterranean^7^ whereas the higher pH variability observed in the NACW has been associated to the influence of biology into the photic zone and changes in evaporation and mixing^46^.

As expected according to the pH_T25_ distribution, *p*CO_2_ levels increased in the LIW (Fig. 2H) with respect to the rest of water masses, with decadal averaged *p*CO_2_ values being 430 ± 17, 407 ± 13 and 376 ± 30 µatm in the LIW, WMDW and NACW respectively. During the monitoring period, surface Atlantic waters were hence undersaturated with respect to atmospheric CO_2_ levels, which gradually rose from 379.8 µatm in 2005 to 400.8 µatm in 2015.

The relatively low pH_T25_ values characterizing Mediterranean waters are taken as an indication of the active organic matter remineralization occurring in the basin^47^. Our AOU data suggest indeed a remarkable oxidation of organic compounds in Mediterranean water masses, as decadal averaged AOU levels were 65 ± 14 μmol kg^−1^ in the LIW and 66 ± 7 μmol kg^−1^ in the WMDW (Fig. 2F). In the case of the NACW, a decadal AOU concentration of 12 ± 22 μmol kg^−1^ was obtained.

*C*_ANT_ distribution in the SoG (Fig. 2I) was also influenced by the pattern of water exchange and circulation. The highest *C*_ANT_ concentrations were attained in the NACW, particularly in the first 100 m depth at the westernmost side of the channel where mixing with the MOW is insignificant (Fig. 2A). Decadal averaged *C*_ANT_ concentrations were 75 ± 16, 71 ± 11 and 52 ± 8 µmol kg^−1^ in the NACW, WMDW and LIW respectively.

It is worth noting the appreciable changes in the vertical profiles of the carbon system parameters westwards of station G2 (Fig. 2D–I), which can be attributed to variation in the water masses proportion brought about by the energetic hydrodynamic feature caused the tidal cycle in the CS area (Fig. 2A–C).

### Archetypal concentrations trends

**Figure 3:**
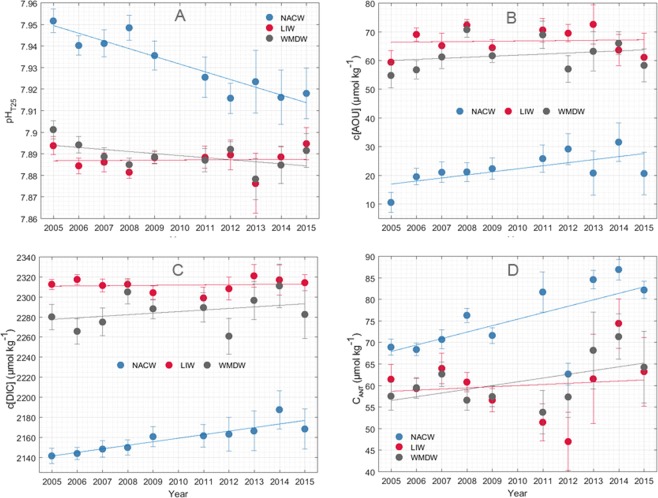
Decadal trends of carbon system parameters in water masses of the Strait of Gibraltar. Annual archetypal concentrations of (**A**) pH_T25_, (**B**) *c*(DIC) (μmol kg^−1^), (**C**) *c*(AOU) (μmol kg^−1^) and (**D**) *C*_ANT_ (μmol kg^−1^) during the 2005–2015 decade in the NACW (blue), LIW (red) and WMDW (grey). Error bars correspond to the standard deviation (SD) of archetypal concentrations of each parameter in all water masses, which were calculated through Eq. 3.

The temporal evolution of archetypal concentrations of the carbon system parameters in the NACW revealed a clear decadal trend (Fig. 3). In particular, pH_T25_ in this water mass showed a marked decrease over the decade 2005–2015 (Fig. 3A), at an annual rate of −0.0036 ± 0.0005 a^−1^ (Table 2). Moreover, AOU and DIC concentrations in Atlantic waters increased annually during the period of study (Fig. 3B,C), at rates of 1.1 ± 0.4 and 3.5 ± 0.6 µmol kg^−1^ a^−1^, respectively (Table 2). Similarly, *C*_ANT_ concentration in the NACW also rose gradually with time, at an annual rate of 1.5 ± 0.6 µmol kg^−1^.

**Table 2:** Rates of variation of carbon system parameters in the water masses present in the Strait of Gibraltar obtained from the temporal evolution of annual archetypal concentrations during the period 2005–2015. SE stands for Standard Error.

	NACW			LIW			WMDW		
	Rate (±SE)	R^2^	*p-*value	Rate (±SE)	R^2^	*p-*value	Rate (±SE)	R^2^	*p-*value
∂c(AOU)/∂t (µmol kg^−1^ a^−1^)	1.1 ± 0.4	0.42	<0.05	0.1 ± 0.5	—	0.86	0.4 ± 0.5	0.06	0.50
∂c(DIC)/∂t (µmol kg^−1^ a^−1^)	3.5 ± 0.6	0.81	<0.05	0.2 ± 0.7	0.01	0.76	1.6 ± 1.5	0.11	0.34
*∂p(*CO_*2*_*)/∂t* (µatm a^−1^)	3.2 ± 0.9	0.60	<0.05	−0.02 ± 0.7	0.02	0.0001	1.2 ± 0.7	0.28	0.11
∂c(*C*_ANT_)/∂t (µmol kg^−1^ a^−1^)	1.5 ± 0.6	0.42	<0.05	0.3 ± 0.7	0.02	0.73	0.9 ± 0.5	0.29	0.11
∂(pH_T25_)/∂t (a^−1^)	−0.0036 ± 0.0005	0.84	<0.05	0.00007 ± 0.0005	—	0.89	−0.0009 ± 0.0005	0.29	0.11
∂ (pH_nobio_)/∂t (a^−1^)	−0.0021 ± 0.0005	0.65	<0.05	0.0001 ± 0.0003	0.03	0.63	−0.0004 ± 0.0005	0.09	0.40

Archetypal concentrations of pH_T25_, AOU and DIC in the LIW were quite stable during the monitoring period and a clear decadal tendency was not observed in any case (Fig. 3). In fact, *c*(AOU) and *c*(DIC) presented the highest annual archetypal levels in relation to those found in the rest of water masses but with negligible annual variation rates (Table 2). On the other hand, a slight temporal increase in the levels of *C*_ANT_ could be detected in the LIW, at a rate of 0.3 ± 0.7 µmol kg^−1^ a^−1^.

In contrast, the WMDW exhibited a noticeable acidification trend over the 2005–2015 decade (Fig. 3A), with pH_T25_ decreasing at a rate of −0.0009 ± 0.0005 a^−1^ (Table 2) although the significance of the statistics was weak. The gradual drop in pH was accompanied by increases in yearly archetypal concentrations of DIC (Fig. 3C) and *C*_ANT_ (Fig. 3D) at rates of 1.6 ± 1.5 µmol kg^−1^ a^−1^ and 0.9 ± 0.5 µmol kg^−1^ a^−1^, respectively (Table 2). The *c*(AOU) also rose during the decade (0.4 ± 0.5 µmol kg^−1^ a^−1^).

Annual archetypal concentrations of *p*CO_2_ were in opposition to the archetypal pH_T25_ levels observed in each water mass (not shown). The upper NACW that was characterized by the lowest decadal average *p*CO_2_ level (Fig. 2H) presented the highest *p*CO_2_ rise during the monitoring period, at a rate of 3.2 ± 0.9 µatm a^−1^ (Table 2). In the WMDW, *p*CO_2_ increased at a rate of 1.2 ± 0.7 µatm a^−1^ whereas the temporal evolution of *p*CO_2_ in the LIW was very smooth (Table 2).

The highest and lowest contribution of the anthropogenic component to the total pH decrease (${\triangle {pH}}_{{\rm{nobio}}})$ was obtained in the NACW and the LIW, respectively (Table 2). In terms of percentage, the ${\triangle {pH}}_{{\rm{nobio}}}$ in the NACW had a weight of approximately 60% of the total annual decreasing pH rate and around 44% in the case of WMDW. Because of the negligible Δ*pH*_*T*2*5*_ measured in the LIW, ${{pH}}_{{\rm{nobio}}}$ in this water mass did not provide any significant information.

## Discussion

Data collected in the SoG during the 2005–2015 decade confirm previous findings indicating that water exchange in the region results in a *C*_ANT_ import from the Atlantic towards the Mediterranean basin^10,11^ as the NACW was always *C*_ANT_ enriched with respect to the MOW. Our periodic observations are also in agreement with the finding that the North Atlantic is the largest ocean sink for anthropogenic carbon^2,48^. The absorption of *C*_ANT_ by the NACW contributed to a noticeable decadal reduction of its pH, with the contribution of the anthropogenic component representing 60% of the total pH decline.

The rise in *C*_ANT_ content over time in the NACW spotted at the SoG occurred at a rate of 1.5 µmol kg^−1^ a^−1^ (Table 2), which matches quite well the decadal rate estimated previously in this water mass^49^. This increment in the quantity of anthropogenic carbon was accompanied by parallel increases in the concentration of DIC and AOU, also suggesting an enhanced organic matter oxidation during the 2005–2015 decade. Before crossing the Strait, the NACW transits through the Gulf of Cádiz (GC in Fig. 1A), a basin characterized by a high primary production^50^. Therefore, regional inputs of organic matter are likely to occur, which could well lead to an intensification in the ecosystem respiration and inorganic carbon accumulation with time. In addition, a considerable discharge of inorganic carbon from the Guadalquivir river estuary to the continental shelf of the GC has been measured^51,52^, which could be accounted as an extra DIC source for the NACW. These external inputs would contribute to the natural component affecting the decadal pH change in this water mass, which represented 40% of the total pH reduction. It is also worth noting that *p*CO_2_ in the NACW rose at a higher rate (3.2 ± 0.9) than the atmospheric CO_2_ annual trend (approximately 2 ppm)^53^. This divergence can be related to the air-sea CO_2_ disequilibrium driven by surface heat fluxes and the balance between the biological carbon uptake and CO_2_ outgassing^46^ that becomes larger with time^54^. Taking into account the atmospheric *p*CO_2_ levels during the monitoring period and an average 90% equilibrium with the anthropogenic fraction^54^, the theoretical pH values in the NACW that would result exclusively from atmospheric CO_2_ uptake were calculated and compared with the estimated ${{pH}}_{{nobio}}$ values. Comparison performed by a Mean Squared Error (MSE) revealed a difference of 0.0001 between both estimates and a mismatch between dissolved and atmospheric *p*CO_2_ concentrations equivalent to 8 years. This finding provides more evidence for local ecosystem drivers of pH fluctuations, as described elsewhere, particularly in coastal regions^17,53^.

Multi-decadal declines of pH in surface Atlantic waters have been observed in the open ocean^5^ yet few studies have quantified the relative importance of biological processes to modulate the pH change over time^15^. The acidification rate calculated here in the NACW from 2005 to 2015 (0.0036 pH a^−1^ Table 2) is of the same order of magnitude than others obtained in distant Atlantic basins although higher^5^ (e.g. 0.0026 and 0.0025 pH a^−1^ in the Irminger Sea and CARIACO basin, respectively). Interestingly, the rate of change of the anthropogenic component (pH_nobio_, 0.0021 pH a^−1^, Table 2) is quite similar to the total rates attained in the Atlantic time series where the rates of dissolved pCO_2_ rise resembled the current atmospheric CO_2_ increase^5,53^. The influence of regional biogeochemical processes on the carbon content of the NACW possibly amplified the temporal pH change and accounted for by the increased acidification trend found with our sustained observations, although this hypothesis remains to be tested.

On the other hand, the decadal rate of pH decrease in the WMDW is comparable to that reported for the Mediterranean finger print in the North Atlantic^55,56^ and lies between the limits established in Mediterranean bottom waters^10^. It can be argued that the pH tendency observed here is not fully supported by the regression statistics. However, it has been recognized that the statistical significance of seawater carbonate chemistry trends in time-series can be weak due to several reasons, such as the irregularity of sampling, non-uniform time intervals between cruises and seasonality in the measurements^5,53^. A further analysis conducted to remove seasonality and identify potential bias of sampling in our databse revealed no seasonal effect on the estimated trends (not shown).

Previous works have already concluded that the MedSea is experiencing an acidification process^9,10,12–14,57^ although the pattern of pH change is not uniform at a basin scale^9,10^, which can be attributable to both the west to east decreasing productivity gradient^58^ and water circulation mechanisms^9,12^.

Each winter, deep waters are formed in both the Eastern and Western Mediterranean. The recently formed waters mix with older and resident water masses, which change their chemical and physical properties; hence, it is not simple to anticipate the effect of water formation events on pH changes. In the particular case of the WMDW, several mechanisms impacting pH at distinct temporal scales must be taken into account, which ultimately complicates the identification of a significant temporal tendency. The WMDW is formed regularly by two processes, deep convection and dense shelf water cascading. The former induces direct downward transport of newly formed organic matter from the surface layer, which is enriched in labile and easily oxidizable material whose decomposition leads to significant CO_2_ release^59,60^. Hence, the WMDW receives a significant amount of *p*CO_2_ from organic matter degradation that is transported down during the formation event. Furthermore, the active ecosystem metabolism occurring in the Alboran sub-basin (AS in Fig. 1) where this water mass resides before leaving the basin^61^ is likely to increase its *p*CO_2_ bulk. In the Alboran Sea, a high primary production in the upper layer^62^ is coupled to a significant export to deep of organic material^58^ and a strong remineralization in the bottom layer^59,63^, which is indeed occupied by the WMDW^61^ (Fig. 2C). Therefore, the WMDW must gain a significant amount of *p*CO_2_ from natural processes before crossing the SoG. Both mechanisms, decomposition of labile organic matter at formation site and the cascading effect during residence time in the Alboran sub-basin would explain the strong biological component (around 60%) of the total pH decline found. The high AOU values measured in the WMDW here and concordant with previous measurements^18^ support this assumption.

Nevertheless, the process of the deep water mass formation in the Gulf of Lions is subject to a marked interannual variability, and thus, pH temporal evolution in the WMDW may not be regular, as evidenced in our time series. Our database identifies a clear pH decline during 2013 (Fig. 3A), which is also evident in the LIW. This particular year marks the occurrence of the Western Mediterranean Transition (WMT) at the SoG^64^. This phenomenon started with a strong WMDW production in the Gulf of Lions during the extreme cold winters of 2005 and 2006 that originated a remarkable deep convection. This deep water enriched in labile organic carbon^18^ arrived to the Alboran Sea and remained in the bottom layer. A subsequent cycle of deep convection during the harsh winters of 2012 and 2013 resulted in newly formed WMDW that was warmer, saltier and denser than the old one^64^ and caused its uplifting and propagation towards the Strait upon arrival in Alboran. The lower pH values observed in the WMDW in 2013 can be interpreted as the footprint of the older (and more acidified) fraction of this water mass after being almost entirely replaced by the more recent fraction. Moreover, it was inferred that the younger WMDW spilled over the SoG in 2015^64^, which would explain the rise in pH observed in this water mass that year (Fig. 3A), corresponding to the signature of a less acidified fraction. The decrease observed in both *C*_ANT_ and *c*(AOU) in 2015 (Fig. 3B,D) is consistent with the presence of a water mass that was rapidly formed and sunk and remained in the AS a lower period of time. This interannual variability in the WMDW time series possibly blurs the identification of statistically significant long term tendencies. Changes in the proportion of fractions of WMDW carrying different levels of both natural and anthropogenic CO_2_ would also account for the discrepancy found between the acidification rate provided here and the one we observed previously in the SoG using three years (from 2012 to 2015) of continuous pH measurements within the MOW^13^. In fact, in the former study considering a shorter period of time, we reported a higher annual pH decline and drastic changes in the proportion of the WMDW during 3 years, which can be related to the influence of the WMT and the subsequent cycle of deep formation events. A thorough investigation of the hydrographic profiles available in our database would certainly contribute to unravel the observed pH variability in the WMDW and strengthen our conclusions but it is not the scope of the present study.

The WMT caused a near-complete renewal of the WMDW^64^ and possibly also some mixing with the LIW, thereby lowering its pH. Blurring of the LIW signal within the MOW has been identified in the SoG and diagnosed through salinity changes^20^, thus mixing between both water masses in the area is a common phenomenon.

Interestingly, during the monitoring period, the LIW exhibited similar pH_T25_ values than those in the WMDW although the temporal evolution of its pH was smoother, as observed in our earlier study^13^. The LIW originates in a distant sub-basin and crosses the entire Mediterranean basin before arriving at the SoG. Therefore, during transit, changes in pH, AOU and *p*CO_2_ are expected to proceed due to physical and biological processes. The LIW route from formation site is complicated as it follows different pathways^65,66^: first it moves westward from the Levantine basin forming two veins, one that flows to the Adriatic Sea and a second one that crosses the Strait of Sicily and enters in the Tyrrhenian. This vein eventually emerges from the Sardinian Channel, reaches the Ligurian Sea, the Gulf of Lions and finally flows towards the Algerian basin^65^. During this journey, changes in biogeochemical parameters, mainly dissolved organic carbon (DOC) and AOU, have been indeed found in response to the different trophic status of the sub-basins crossed^18^. In particular, the LIW passes by areas of strong organic matter remineralization that cause minimum contents of DOC and maximum levels of AOU^18^. The *c*(AOU) measured in the LIW during the 2005–2010 decade (within the range 60–70 µmol kg^−1^) are possibly the final result of all the biological changes experienced during transit, which modified original pH as well. Furthermore, as the LIW remains at intermediate depths in the SoG (Fig. 2B), Algerian and Alboran sub-basins^61^ the additional contribution of cascading processes to its *p*CO_2_ bulk cannot be totally neglected. Therefore, re-ventilation mechanisms suffered by the LIW, vertical transport of carbon by diffusion and mixing with the WMDW must alter its original biogeochemical characteristics and consequently lower its pH.

In fact, when the ages of the WMDW and the LIW are estimated, similar values are obtained and equivalent to 32 ± 8 and 34 ± 8 years, respectively (MSE < 0.0001). Alternatively, age calculation based on available CFC data^39^ yields estimates of 18 years for the WMDW and 21 years for the LIW. Although there is no consensus about the exact ages of Mediterranean water masses and discrepant values have been provided^67,68^, it is clear that our calculated ages for the LIW are lower than that expected for a water mass that has crossed the entire basin. Actually, the age of the LIW in the Western basin has been proposed to be in the range of 82–120 years^67^.

These findings and the fact that appreciable *C*_ANT_ levels were measured in the LIW support the occurrence of re-ventilaton and/or mixing with younger water masses. If we consider that the LIW currently spotted at the SoG corresponds to a water mass that sank originally in the Eastern basin and lost contact with the atmosphere roughly one century ago, it is plausible to assume that its *C*_ANT_ content would be negligible, as it was exposed to a very low atmospheric CO_2_ concentration upon formation (resembling the CO_2_ pre-industrial level). During transit, its position within the water column prevented any new contact with the atmosphere, thus hampering the absorption of the rising atmospheric CO_2_. Therefore, the LIW must gain anthropogenic carbon by other processes, and the most likely candidates are re-ventilation and mixing with more recently formed water masses, such as the Cretan Intermediate Water (CIW) in the Ionian Sea^18^ and the WMDW in the SoG^20^. This would alter the original biogeochemical characteristics of this water mass and in turn, estimations of its age and anthropogenic signals.

As the drivers of anthropogenic forcing and natural variability are not entirely constrained and their signals cannot be discerned at a statistically significant level, identification of acidification trend in the LIW would require a longer time period of periodic observations, as it has been recently shown in a number of time series^53^.

## Conclusions

Our study evidences the presence of an anthropogenic impact on two major Mediterranean water masses, as appreciable *C*_ANT_ levels were measured in the WMWD and the LIW using 11 years of sustained observations at the Strait of Gibraltar. However, a clear acidification trend could only be identified in the former, with the contribution of the anthropogenic driver to the total pH change representing 40% of the decadal decline. As the LIW is an old water mass with no contact with the current atmosphere, its *C*_ANT_ content may be attributable to further contamination during transit across the basin from formation site. Longer monitoring period than that encompassed by our study and observations in the Eastern Mediterranean are required to detect long term pH variations in the LIW and constrains the relative importance of the natural and anthropogenic signals on its pH evolution.

It is plausible that the acidification phenomenon in the MedSea could be exacerbated in the future, as our assessment also shows that the incoming Atlantic water suffered a marked acidification trend over the decade. In this case, reduction in the pH of the NACW was dominated by the anthropogenic component, which accounted for by 60% of the total decrease. Regional biogeochemical processes might amplify natural pH variability in this water mass, resulting in higher acidification rates in relation to others observed in surface Atlantic waters.

Regardless of the relative contribution of the drivers affecting pH decline in Atlantic and Mediterranean waters, our findings are in support of the current concerns regarding the fate of marine ecosystems due to the increase in acidity of the seawater. Particularly, iconic habitats of cold water corals of the North Atlantic^69^ and Mediterranean Sea^70^ are expected to be highly impacted by the phenomenon, which is now corroborated by our assessment.

Hence, even though further work should be directed at improving upon our estimates by increasing the length of sustained observations, our decadal records still provide a starting point for future calculations of ocean acidification trends in Mediterranean waters and reveal the importance of long time series in this marginal sea.

## Data Availability

The dataset generated during and/or analysed during the current study are available from the corresponding author upon reasonable request.
